# Takotsubo stress cardiomyopathy following explantation of sEEG electrodes

**DOI:** 10.1002/epi4.12452

**Published:** 2021-02-15

**Authors:** Pamela Sarkar, John Graby, Paul Walker, Leyla Osman, Marcus Bradley, Marcus Likeman, David R. Sandeman, Kasia A. Sieradzan, Claire M. Rice

**Affiliations:** ^1^ Southmead Hospital North Bristol NHS Trust Bristol UK; ^2^ Queen Elizabeth Hospital Edgbaston, Birmingham UK; ^3^ Clinical Neurosciences, Translational Health Sciences University of Bristol Bristol UK

**Keywords:** intracerebral hemorrhage, stereoelectroencephalography, Takotsubo stress cardiomyopathy

## Abstract

**Objective:**

Takotsubo stress cardiomyopathy is characterized by dysfunction of the left ventricle of the heart including apical ballooning and focal wall‐motion abnormalities. Although reported in association with seizures and intracerebral hemorrhage, there are no studies reporting its occurrence in patients having stereoelectroencephalography (sEEG).

**Methods:**

A 38‐year‐old lady with no prior history of cardiac disease experienced sudden onset chest pain and acute left ventricular failure 4 hours following explantation of stereoelectroencephalogram electrodes.

**Results:**

A small parenchymal hematoma related to the right posterior temporal electrode had been noted postelectrode insertion but was asymptomatic. Focal‐onset seizures from nondominant mesial temporal structures were recorded during sEEG. Following the presentation with LVF, new‐onset anterolateral T‐wave inversion with reciprocal changes in leads II, III, and aVF was noted on electrocardiogram (ECG) and the chest X‐ray findings were consistent with pulmonary edema. Echocardiography demonstrated hypokinesis of the cardiac apex and septum consistent with Takotsubo stress cardiomyopathy.

**Significance:**

Awareness of the possible complication of Takotsubo stress cardiomyopathy is required in an epilepsy surgery program.

**Key Points**

- Takotsubo stress cardiomyopathy (TTS) or “stress‐induced cardiomyopathy” has been reported in the context of intracerebral hemorrhage and seizures
- We report the first case of TTS occurring following sEEG explantation
- Awareness of the possibility of TTS is required in an epilepsy surgery program

## 1 INTRODUCTION

We present the case of a 38‐year‐old lady with no prior history of cardiac disease who experienced sudden onset chest pain and acute left ventricular failure 4 hours following explantation of stereoelectroencephalogram electrodes. Echocardiography demonstrated hypokinesis of the cardiac apex and septum consistent with Takotsubo stress cardiomyopathy which has not been previously reported in the context of stereoelectroencephalography. The aim of this case report was to raise awareness of the potential complication of Takotsubo stress cardiomyopathy in an epilepsy surgery program.

## 2 CASE REPORT

**FIGURE 1:**
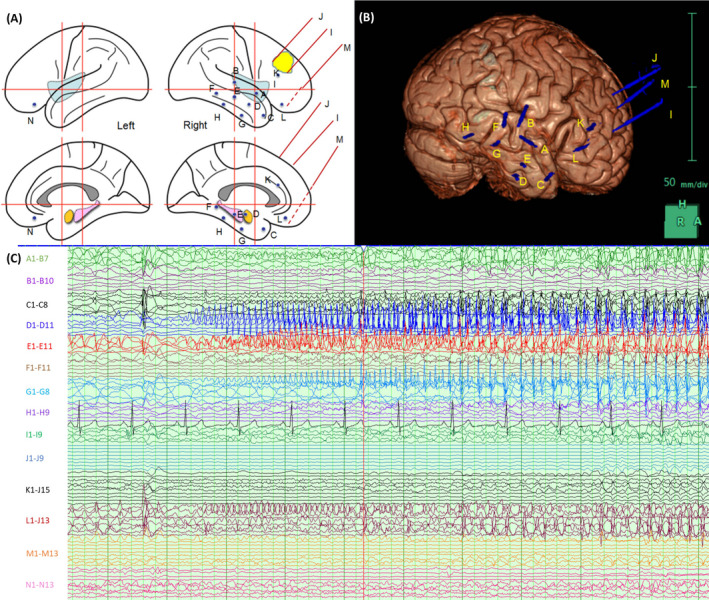
sEEG electrode placement and representative seizure. sEEG electrode placement was as illustrated (A and B): A‐R superior temporal 1 (deep contacts abnormal area insula)—8 contacts; B‐R superior temporal 2 (caudal, deep contacts post insula)—10 contacts; C‐R temporal pole (via T2, deep contacts in mesial ant temporal cortex)—8 contacts; D‐R amygdala (via T2 into amygdala)—12 contacts; E‐R hippocampus anterior (via T2 into R hippocampus ant)—12 contacts; F‐R temporal post (via posterior T2 into basal temporal lobe (fusiform)—12 contacts; G‐R temporal basal 1 (via T3, rostral exploring basal temp lobe)—10 contacts; H‐R temporal basal 2 (via T3, caudal exploring basal temp lobe)—10 contacts; I‐R frontal oblique 1 (rostral margin of lesion R middle frontal gyrus)—10 contacts; J‐R frontal oblique 2 (caudal margin of lesion R middle frontal gyrus)—10 contacts; K‐R frontal below the margin of scarring in F2, deep contact ant cingulate—10 contacts; L‐R orbitofrontal orthogonal—15 contacts; M‐R orbitofrontal oblique—into R gyrus rectus—15 contacts; N‐L orbitofrontal—(orthogonal or oblique targeting L gliosis in L gyrus rectus)—15 contacts; sEEG (C) demonstrates evolving mesial temporal discharge in the amygdala and anterior hippocampus spreading to the basal temporal and orbitofrontal cortices

A 38‐year‐old, right‐handed lady developed chest pain and acute pulmonary edema 4 hours following explantation of stereoelectroencephalogram (sEEG) electrodes. She had poorly controlled, post‐traumatic right temporal lobe epilepsy but no additional past medical history or risk factors for cardiovascular disease. MRI brain demonstrated multiple areas of gliosis, particularly laterally and anteromedially in the right temporal lobe, the right, and to a lesser degree, left orbitofrontal cortex, with focal white matter loss in the corpus callosum. Video‐telemetry was consistent with focal‐onset, nondominant mesial temporal or orbitofrontal lobe seizures; semiology consisted of right upper limb and oral motor automatisms preceded by prolonged aura, including olfactory and autonomic features. Generalized convulsive seizures were not observed during this period of recording. Robotic sEEG (Renishaw Neuromate) was undertaken to further localize seizure onset; 13 electrodes were implanted in the right hemisphere in a frontotemporal distribution and 1 in the left orbitofrontal region (Figure 1A,B). Routine postinsertion CT demonstrated a small parenchymal hematoma related to the right posterior temporal electrode ((electrode F); entry via middle temporal gyrus, deep contacts in the fusiform gyrus) which was asymptomatic. Over 9 days, a total of 14 stereotyped seizures were recorded (Figure 1C); all initiated from the right mesial temporal structures starting in the amygdala with rapid spread to the hippocampus and right temporal pole. Apparently uneventful explantation occurred at day 10 on the ward. This was several hours after the last recorded seizure, and prophylactic enoxaparin had been withheld for 48 hours.

**FIGURE 2:**
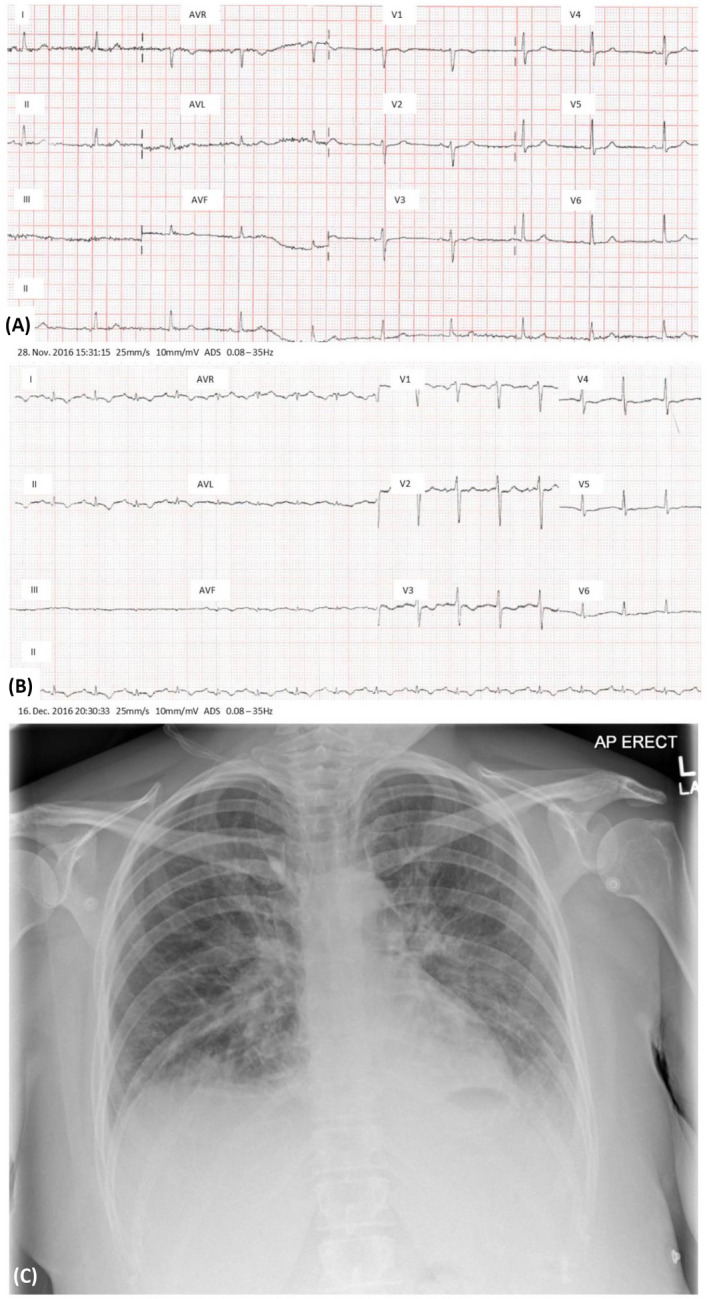
ECG changes and chest X‐ray during presentation with acute chest pain. ECGs on admission (A) and during the acute episode (B) demonstrate new antero‐lateral T wave inversion with reciprocal changes in leads II, III and aVF. The chest X‐ray (C) appearances are consistent with pulmonary edema

Four hours postexplantation during which time seizures did not occur, the patient experienced severe central chest pain at rest with radiation to the neck and associated dyspnea, diaphoresis, and nausea. Pain responded to glyceryl trinitrate and morphine. She denied headache. Oxygen (O_2_) saturations were 73% on air, improving to 98% with 3 L O_2_. There was hemodynamic instability, sinus tachycardia (rate 125/min) and episodic hypotension (minimum systolic 70 mmHg). Cardiorespiratory examination was consistent with pulmonary edema secondary to acute left ventricular (LV) failure, and there were no focal neurological findings or seizures. Electrocardiogram demonstrated new‐onset anterolateral T‐wave inversion with reciprocal changes in leads II, III, and aVF (Figure 2A,B). Chest X‐ray findings confirmed pulmonary edema (Figure 2C). Intravenous frusemide was administered, and, given concern regarding the possibility of non‐ST elevation myocardial infarction (NSTEMI), dual antiplatelet therapy was commenced.

**FIGURE 3:**
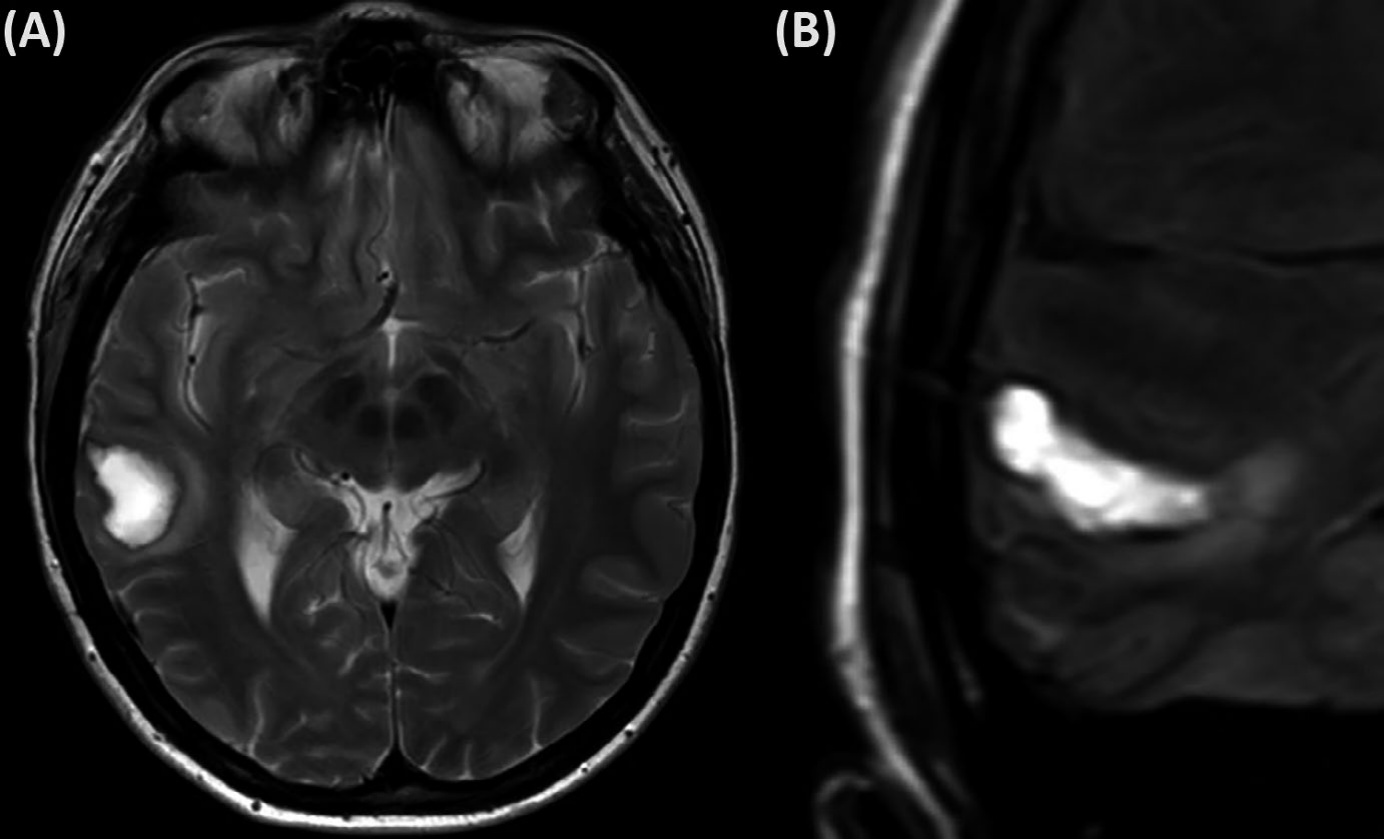
Neuroimaging changes consistent with intracranial hemorrhage. T2 MRI (A) and FLAIR (B) of the brain demonstrate a hematoma in the right temporal lobe

Clinical improvement with resolution of chest pain and dyspnea occurred over 24 hours. Troponin level rose from 13 to 33 ng/L. ECG changes resolved, and the coronary angiogram was normal. Antiplatelet therapy was discontinued at 48 hours. Transthoracic echocardiogram revealed evidence of moderate LV systolic dysfunction (estimated ejection fraction 40%–45%) and hypokinesis of the cardiac apex as well as the anterior and anteroseptum. The right temporal lobe hematoma was again noted on MRI brain at 2 days postexplantation along with right frontal pole edema (Figure 3A,B) but there was no extension of the hematoma when compared with the initial postexplantation CT scan. TTE demonstrated improvement in LV systolic function at 4 weeks and near normal function at 6 months.

Following initial concern regarding the possibility of NSTEMI, the diagnosis was revised to takotsubo stress cardiomyopathy (TTS). TTS, also known as “stress‐induced cardiomyopathy” and “broken heart syndrome,” is characterized by LV dysfunction with regional wall abnormalities including apical ballooning and focal wall‐motion abnormalities; the end‐systole radiological appearance of the left ventricle is said to resemble a Japanese octopus trap (“takotsubo”). TTS occurs more often in postmenopausal females and at times of emotional and physical stress.^1^ The pathogenesis remains uncertain although sympathetic stimulation is key^1^ and a link between the insular cortex and central autonomic network recognized.^2^ Acute neurological diseases including subarachnoid hemorrhage, stroke, and seizures are well‐recognized precipitants for TTS,^1^ and an association between TTS and sudden unexplained death in epilepsy (SUDEP) has been proposed.^1^ TTS has not previously been recorded in the context of sEEG.

Level C evidence for treatment of TTS is available, and management is generally based on guidelines for treatment of acute coronary artery syndrome with particular consideration given to beta‐blockers in view of association with elevated catecholamine levels, diuretics for pulmonary edema, and nitroglycerin in LV failure.^3^

Intracranial hemorrhage (ICH) is well recognized to be a precipitant for TTS, and ICH has been reported to occur in approximately 1% patients undergoing sEEG.^4^ The presence of temporal lobe hemorrhage could therefore have been a trigger for TTS. Takosubo syndrome has also been reported several days after convulsive and nonconvulsive seizures,^5^ so seizure activity may also have been a contributory factor. While we cannot categorically state that explantation induced TTS in our patient, we consider the timing suggestive although the association has not, to the best of our knowledge, been reported previously. Furthermore, the multiple potential triggers may have contributed to the presentation.

Given the potential for overlapping risk factors of seizures and ICH, this case highlights the importance of awareness of TTS in the context of an epilepsy surgery program. We advise early involvement of cardiologists in the care of patients suspected of having TTS and, if clinically safe to do so, prompt neuroimaging to exclude ICH.

## CONFLICTS OF INTEREST

None of the authors has any conflict of interest to declare. We confirm that we have read the Journal's position of issues involved in ethical publication and affirm that this report is consistent with those guidelines.

## References

[1] Ghadri JR , Wittstein IS , Prasad A , Sharkey S , Dote K , Akashi YJ , et al. International expert consensus document on Takotsubo Syndrome (Part I): clinical characteristics, diagnostic criteria, and pathophysiology. Eur Heart J. 2018;39(22):2032–46. 29850871 10.1093/eurheartj/ehy076 PMC5991216

[2] Ranieri M , Finsterer J , Bedini G , Parati EA , Bersano A . Takotsubo Syndrome: clinical features, pathogenesis, treatment, and relationship with cerebrovascular diseases. Curr Neurol Neurosci Rep. 2018;18(5):20. 29569186 10.1007/s11910-018-0833-7

[3] Ghadri JR , Wittstein IS , Prasad A , Sharkey S , Dote K , Akashi YJ , et al. International Expert Consensus Document on Takotsubo Syndrome (Part II): diagnostic workup, outcome, and management. Eur Heart J. 2018;39(22):2047–62. 29850820 10.1093/eurheartj/ehy077 PMC5991205

[4] Mullin JP , Shriver M , Alomar S , Najm I , Bulacio J , Chauvel P , et al. Is SEEG safe? A systematic review and meta‐analysis of stereo‐electroencephalography‐related complications. Epilepsia. 2016;57(3):386–401. 26899389 10.1111/epi.13298

[5] Stollberger C , Sauerberg M , Finsterer J . Immediate versus delayed detection of Takotsubo syndrome after epileptic seizures. J Neurol Sci. 2019;15(397):42–7. 10.1016/j.jns.2018.12.005 30583237

